# Comparison of brain-derived versus conventional pTau biomarkers in CSF and plasma for detecting Alzheimer’s disease

**DOI:** 10.1093/braincomms/fcag376

**Published:** 2026-10-07

**Authors:** Lydia Trudel, Joseph Therriault, Valentina Ghisays, Arthur C Macedo, Serge Gauthier, Paolo Vitali, Andrea L Benedet, Henrik Zetterberg, Yi Su, Alpana Singh, Tainá Marques, Marisa N Denkinger, Nicholas T Seyfried, Allan I Levey, Michael W Weiner, Kendall Van Keuren-Jensen, Eric M Reiman, Nicholas J Ashton, Pedro Rosa-Neto

**Affiliations:** Translational Neuroimaging Laboratory, Montreal Neurological Institute, Montreal, Quebec H3A 2B4, Canada; Department of Neurology and Neurosurgery, Faculty of Medicine, McGill University, Montreal, Quebec H3A 2B4, Canada; Translational Neuroimaging Laboratory, Montreal Neurological Institute, Montreal, Quebec H3A 2B4, Canada; Department of Neurology and Neurosurgery, Faculty of Medicine, McGill University, Montreal, Quebec H3A 2B4, Canada; Banner Alzheimer’s Institute, Phoenix, AZ 85006, USA; Translational Neuroimaging Laboratory, Montreal Neurological Institute, Montreal, Quebec H3A 2B4, Canada; Department of Neurology and Neurosurgery, Faculty of Medicine, McGill University, Montreal, Quebec H3A 2B4, Canada; Translational Neuroimaging Laboratory, Montreal Neurological Institute, Montreal, Quebec H3A 2B4, Canada; Department of Neurology and Neurosurgery, Faculty of Medicine, McGill University, Montreal, Quebec H3A 2B4, Canada; Translational Neuroimaging Laboratory, Montreal Neurological Institute, Montreal, Quebec H3A 2B4, Canada; Department of Neurology and Neurosurgery, Faculty of Medicine, McGill University, Montreal, Quebec H3A 2B4, Canada; Department of Psychiatry and Neurochemistry, Institute of Neuroscience and Physiology, the Sahlgrenska Academy, University of Gothenburg, Möldnal, 413 90, Sweden; Department of Psychiatry and Neurochemistry, Institute of Neuroscience and Physiology, the Sahlgrenska Academy, University of Gothenburg, Möldnal, 413 90, Sweden; Clinical Neurochemistry Laboratory, Sahlgrenska University Hospital, Gothenburg 413 45, Sweden; Department of Neurodegenerative Disease, UCL Queen Square Institute of Neurology, London WC1N 3BG, UK; UK Dementia Research Institute at UCL, London NW1 3BT, UK; Hong Kong Center for Neurodegenerative Diseases, Hong Kong, China; Wisconsin Alzheimer’s Disease Research Center, University of Wisconsin School of Medicine and Public Health, University of Wisconsin-Madison, Madison, WI 53792, USA; Banner Alzheimer’s Institute, Phoenix, AZ 85006, USA; Banner Sun Health Research Institute, Sun City, AZ 85351, USA; Banner Sun Health Research Institute, Sun City, AZ 85351, USA; Banner Sun Health Research Institute, Sun City, AZ 85351, USA; Department of Neurology, Emory University School of Medicine, Atlanta, GA 30322, USA; Goizueta Alzheimer’s Disease Research Center, Emory University School of Medicine, Atlanta, GA 30329, USA; Center for Imaging of Neurodegenerative Diseases, Northern California Institute for Research and Education (NCIRE), San Francisco, CA 94121, USA; Departments of Radiology, Medicine, Psychiatry, & Neurology, University of California San Francisco, San Francisco, CA 94121, USA; Neurogenomics Division, TGen, Phoenix, AZ 85004, USA; Banner Sun Health Research Institute, Sun City, AZ 85351, USA; Banner Alzheimer’s Institute, Phoenix, AZ 85006, USA; Department of Psychiatry and Neurochemistry, Institute of Neuroscience and Physiology, the Sahlgrenska Academy, University of Gothenburg, Möldnal, 413 90, Sweden; Banner Sun Health Research Institute, Sun City, AZ 85351, USA; Translational Neuroimaging Laboratory, Montreal Neurological Institute, Montreal, Quebec H3A 2B4, Canada; Department of Neurology and Neurosurgery, Faculty of Medicine, McGill University, Montreal, Quebec H3A 2B4, Canada; Peter O’Donnell Jr. Brain Institute (OBI), University of Texas Southwestern Medical Centre (UTSW), Dallas, TX 75235, USA

**Keywords:** biomarkers, Alzheimer’s disease, brain-derived, phosphorylated tau

## Abstract

Brain-derived phosphorylated tau species are postulated to improve the detection of Alzheimer’s disease pathology by mitigating non-specific increases. However, their true diagnostic performance, relative to conventional CSF and plasma phosphorylated tau assays, remains uncharacterized. The objective was to compare the diagnostic performance of brain-derived and conventional phosphorylated tau assays across multiple analytes (phosphorylated tau 181, phosphorylated tau 217, phosphorylated tau 231) in CSF and plasma, and to examine biomarker differences across amyloid-β status and cognitive groups. This study included 1098 participants with amyloid-PET imaging and CSF samples from the Alzheimer’s Disease Neuroimaging Initiative, and a subset of 925 individuals with plasma samples. Diagnostic performance of brain-derived and conventional phosphorylated tau assays was assessed by receiver operating characteristic curves, with calculation of area under the curve, sensitivity, specificity and optimal cutpoints. Associations with amyloid-β burden were evaluated using Spearman correlations, and fold-changes between amyloid-PET-positive and amyloid-PET-negative participants were computed in cognitively unimpaired and cognitively impaired subgroups with 95% confidence intervals from 2000 bootstrap resamples. Brain-derived phosphorylated tau assays showed significantly higher diagnostic performance than conventional phosphorylated tau assays at the same phosphorylation site in both CSF and plasma in the overall sample and in cognitively impaired individuals, except for CSF phosphorylated tau 217. While CSF brain-derived phosphorylated tau correlations with amyloid-PET were similar to conventional CSF assays, pairwise comparisons using DeLong’s test revealed significantly higher diagnostic accuracy for brain-derived assays. Receiver operating characteristic performance was generally better in cognitively impaired participants than in cognitively unimpaired participants. Additionally, using a three-range method with cutpoints corresponding to 90% sensitivity and 90% specificity, plasma brain-derived phosphorylated tau 181, phosphorylated tau 217 and brain-derived phosphorylated tau 217 had <20% of participants in the intermediate range overall and in cognitively impaired individuals. In summary, brain-derived phosphorylated tau assays improve detection of amyloid-β positivity relative to conventional assays, especially in plasma, and provide robust differentiation of amyloid-PET-positive and amyloid-PET-negative participants. If these findings are confirmed using fully automated diagnostic platforms that are intended for use in clinical settings, brain-derived phosphorylated tau assays may support future blood-based biomarker triage workflows for identifying Alzheimer’s disease.

## Teaser

Trudel *et al*. report that brain-derived plasma phosphorylated tau (pTau) biomarkers, particularly pTau217, outperform conventional plasma and cerebrospinal fluid measures for identifying amyloid-positron emission tomography positivity. Brain-derived enrichment reduced intermediate classifications and improved diagnostic performance, supporting its potential clinical utility in Alzheimer disease.

## Graphical Abstract

**Graphical Abstract:**
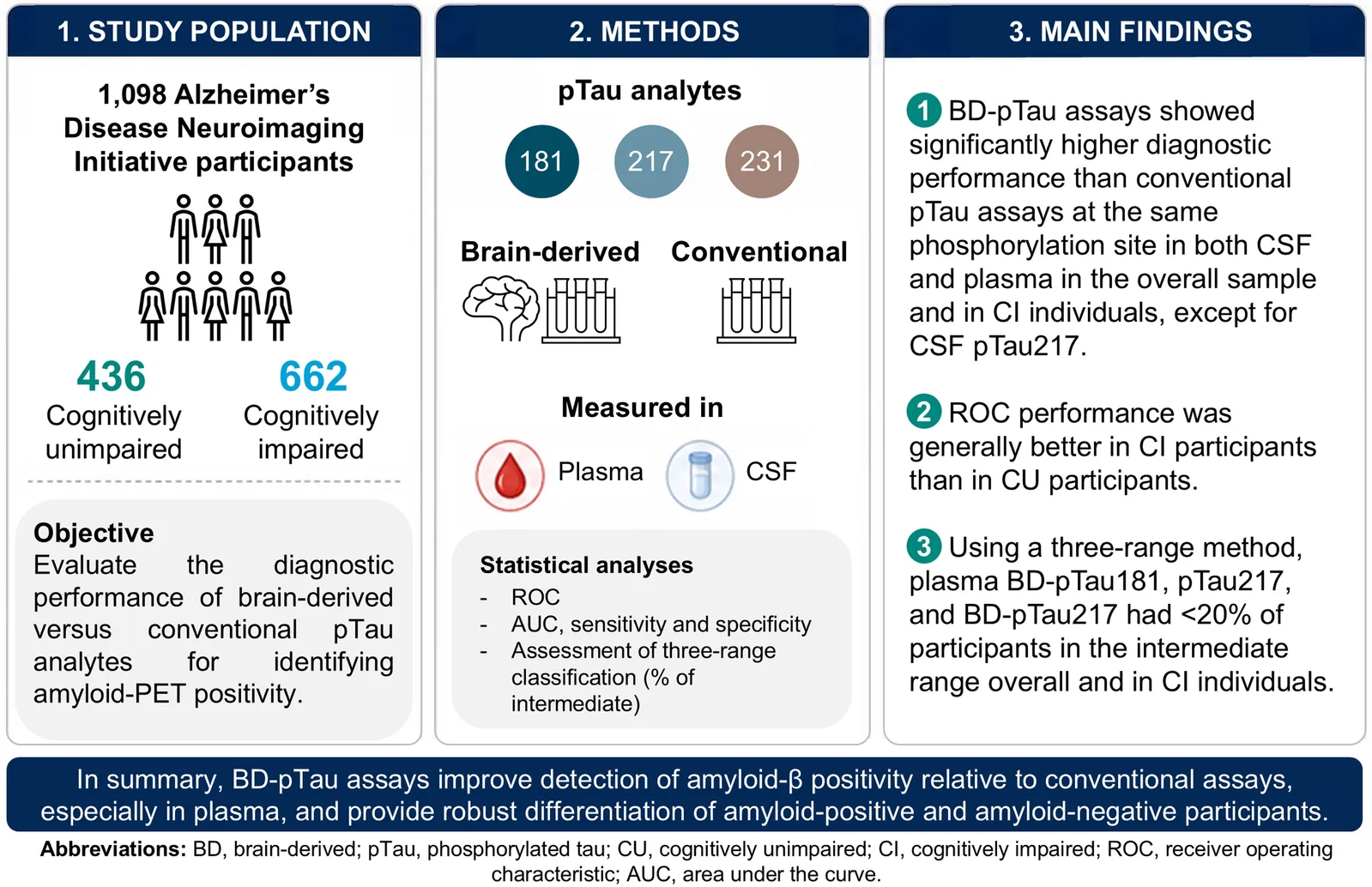

## Introduction

Phosphorylated tau (pTau) has fundamentally transformed the biological diagnosis of Alzheimer’s disease (AD), enabling sensitive detection of AD-related pathology using minimally invasive blood-based assays.^1-3^ While conventional plasma pTau measures have demonstrated strong diagnostic performance,^4-10^ often favouring superior negative predictive value, their interpretation may be complicated by contributions from peripheral sources, which can lead to elevated levels in other conditions such as amyotrophic lateral sclerosis.^11^ These limitations highlight the need for assays that more specifically capture tau originating from the central nervous system, particularly if blood-based biomarkers are to be deployed in broader clinical settings outside specialized memory clinics, where the unselected nature of the patient population will require straightforward interpretation for guiding diagnosis and management.

Recently, brain-derived (BD) pTau assays have been developed to enrich for tau species originating from the central nervous system, with the aim of increasing specificity for AD-related pathology.^12^ This is achieved by partnering the phosphorylated tau capture antibody with a secondary antibody raised to specifically exclude the large peptide insert resulting from the transcription of an extra exon (exon 4a) of the *MAPT* gene or *Big Tau*. This hypothetically reduces confounding signal from peripheral sources, thereby yielding measures that are expected to more closely reflect CNS AD-related pathology. These advances have been facilitated by ultrasensitive proteomic platforms, which enable high-specificity detection of low-abundance, CNS-relevant proteins in both plasma and CSF through effective suppression of background signal.^13^

In the present study, we compared three pTau analytes (pTau181, pTau217 and pTau231) measured in both CSF and plasma using BD-pTau and conventional pTau assays to evaluate their relative performance for identifying elevated amyloid-positron emission tomography (PET) signal, and examined how these associations varied between cognitively unimpaired (CU) and cognitively impaired individuals (CI).

## Materials and methods

### Study population

This cross-sectional study included 1098 participants from the Alzheimer’s Disease Neuroimaging Initiative (ADNI) database.^14^ ADNI is a multicentre, longitudinal study launched in 2004 with the primary goal of developing clinical, imaging, genetic, and fluid biomarkers for the early detection and tracking of AD. Information regarding inclusion/exclusion criteria of the ADNI cohort are available at https://adni.loni.usc.edu/data-samples/. Our study included CU individuals, as well as participants with mild cognitive impairment (MCI) or dementia, all with available CSF pTau measurements. Plasma pTau data were available for a subset of 925 participants. Diagnostic classifications were assigned by ADNI at each visit using standardized clinical criteria, including interviews, neuropsychological testing, and functional assessments. All participants provided written informed consent, and ADNI protocols were approved by the institutional review boards at each participating site.

### Fluid biomarkers

Detailed methods for fluid biomarker quantification are provided in the Methods in Supplementary Materials. Plasma and CSF samples were analysed using the Alamar NULISAseq™ CNS Disease Panel at the Michael T. Zuendel Family Laboratory at Banner Sun Health Research Institute.^15^ Prior to assay, samples were thawed and centrifuged at 2200 × g for 10 min to remove particulate matter. A total of 25 µl of supernatant from each sample was transferred to 96-well plates for analysis. Samples were assayed using the NULISAseq™ CNS Disease Panel 120 (Alamar Biosciences),^13^ a brain-derived panel comprising 131 analytes. APOE4 was excluded from plasma analyses, while APOE and CRP were excluded from CSF analyses. Immunocomplex formation was achieved using paired oligonucleotide-conjugated antibodies specific to each target protein. Subsequent assay steps including oligo-dT bead capture, release, secondary capture using streptavidin beads, and ligation were performed on the automated Alamar ARGO HT system according to the manufacturer’s protocol. DNA reporter libraries containing both target-specific molecular identifiers (TMIs) and sample-specific molecular identifiers (SMIs) were pooled, amplified by polymerase chain reaction (PCR), purified, and sequenced using the Illumina Novaseq X. Assay performance was monitored using pooled plasma, internal control reporters, inter-plate controls, and negative controls. Target counts were first normalized to internal controls and then to inter-plate control medians. Values were rescaled, log_2_-transformed, and reported as NULISA Protein Quantification (NPQ) units, which reflect relative protein abundance and log_2_ fold changes between samples or groups. All biomarker concentrations are reported as NPQ values, which represent normalized, log_2_-transformed measures of protein abundance.

### Neuroimaging

Cerebral amyloid-β burden was assessed using PET imaging acquired as part of the ADNI protocol,^16^ using either [^18^F]florbetapir or [^18^F]florbetaben. PET images were centrally preprocessed by ADNI using validated pipelines, including frame realignment, averaging, spatial normalization to a common template, and intensity normalization.  8 mm SUVRs were transformed to 6 mm equivalent using ADNI-derived conversion equations, which are highly correlated with 6 mm SUVRs (*R*^2^ ≥ 0.96).^17^ Global cortical amyloid-β burden was quantified using standardized uptake value ratios (SUVR), computed across predefined cortical regions known to accumulate amyloid-β pathology, including frontal, temporal, parietal, and cingulate cortices. Image acquisition followed standardized ADNI procedures to ensure harmonization across sites and tracers. To facilitate comparability across tracers, SUVR values were converted to Centiloid units using established transformation procedures.^18^ Amyloid-β positivity was determined using the ADNI *AMYLOID_STATUS* variable, which applies thresholds ([^18^F]florbetapir: 1.11 or [^18^F]florbetaben: 1.08), respectively, established by the ADNI PET Core^19^) to the cortical summary SUVR normalized by whole cerebellum.^17^

### Statistical analysis

Associations between pTau biomarkers and Centiloids were assessed using Spearman correlations, performed separately for each analyte and assay type (i.e. brain-derived versus conventional). To evaluate the diagnostic performance of plasma and CSF pTau biomarkers in distinguishing amyloid-positive from amyloid-negative individuals, receiver operating characteristic (ROC) curves were generated, and the corresponding area under the curve (AUC), optimal sensitivity, specificity, and cutpoints were determined. ROC analyses and AUC comparisons were performed for the entire sample as well as separately in CU and CI participants. Pairwise comparisons of AUCs between BD and conventional assays were performed using DeLong’s test to assess statistical significance. *P*-values were adjusted for multiple comparisons using the Benjamini–Hochberg false discovery rate procedure across the comparisons. In addition, a three-range classification method was applied using cutpoints corresponding to 90% sensitivity and specificity, with a secondary analysis using 95% thresholds as a sensitivity check, and the proportion of participants falling in the intermediate range was computed for each biomarker. Finally, fold-change analyses were conducted to quantify the magnitude of biomarker elevation in amyloid-PET-positive (A+) versus amyloid-PET-negative (A−) participants, performed separately in CU and CI subgroups, with 95% confidence intervals derived from 2000 bootstrap resamples of the fold-change ratios. Statistical analyses were performed in R v.2024.04.2,^20^ and significance was set at *P* < 0.05. In an exploratory analysis, we evaluated and compared diagnostic performance using ROC analyses in a subsample of 142 participants (69 CU, 73 CI) with head-to-head plasma pTau217 measurements across five assay platforms (NULISA, AlzPath, Janssen, Fujirebio and C2N) with plasma BD-pTau217. Methods for fluid quantification for all assays have been described previously.^21^

## Results

### Demographic characteristics

Of the 1098 individuals included in this study, 553 were women (50.4%) and 545 were men (49.6%). Mean (SD) age was 72.4 (7.27) years. 436 participants were CU (39.7%), and 662 participants were CI (60.3%). 32.3% of CU individuals were A+, and 60.7% of CI individuals were A + . Other demographic and clinical characteristics are reported in Table 1.

**Table 1:** Demographic information of the study sample

	CU (*N* = 436)	MCI (*N* = 515)	Dementia (*N* = 147)	Overall (*N* = 1098)
Age, mean (SD), y	72.4 (6.43)	71.9 (7.51)	74.2 (8.49)	72.4 (7.27)
Female, no. (%)	266 (61.0)	228 (44.3)	59 (40.1)	553 (50.4)
Education, mean (SD), y	16.8 (2.35)	16.2 (2.66)	15.8 (2.60)	16.4 (2.55)
CDR-SB	0.0471 (0.175)	1.45 (0.901)	4.46 (1.62)	1.30 (1.65)
Centiloids, mean (SD)	20.2 (34.9)	39.9 (47.8)	82.2 (46.0)	37.8 (47.2)

### Distribution of pTau biomarkers across diagnostic groups

We first measured CSF and plasma levels of both BD and conventional pTau181, pTau231 and pTau217 across diagnostic groups (Fig. 1). Across all analytes and sample types, A + participants consistently exhibited higher biomarker levels than A− participants, including in CU individuals. Furthermore, CSF BD-pTau assays closely mirrored conventional CSF assays. Plasma BD assays tracked conventional plasma pTau levels closely, though the range of values was slightly smaller, resulting in less variability within each diagnostic groups.

**Figure 1:**
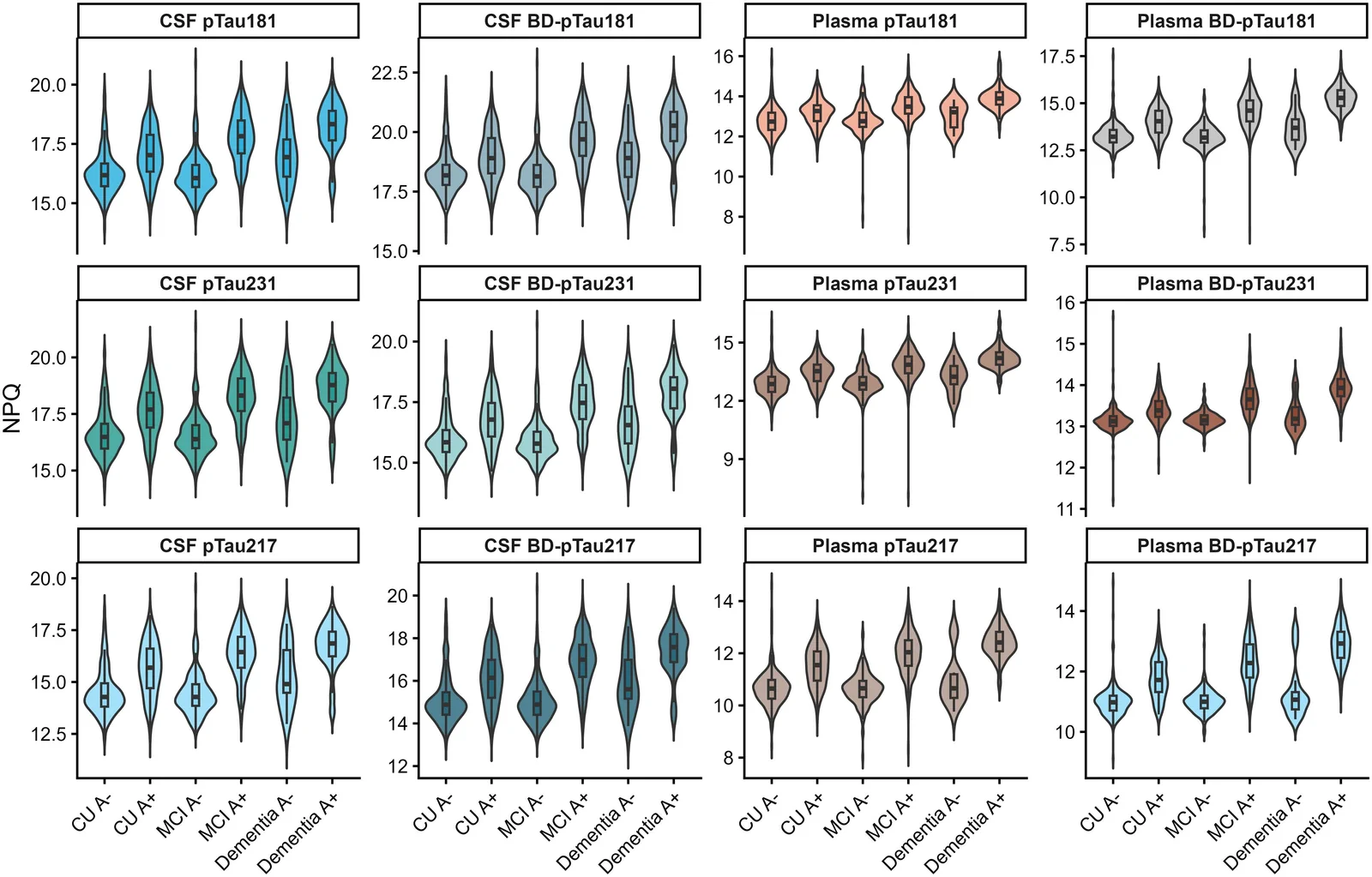
**Summary statistics of CSF and plasma pTau biomarkers across diagnostic groups.** Violin plots showing the distribution of biomarker levels for each diagnostic group, with boxplots indicating median and interquartile range. Panels are faceted by biomarker, with rows ordered by analyte (pTau181, pTau231, pTau217) and columns by sample type (CSF, CSF BD, plasma, plasma BD). The experimental unit was the individual participant, with each participant contributing one measurement per biomarker. MCI, mild cognitive impairment; NPQ, NULISA protein quantification units.

### Fold-change comparison of conventional and BD-pTau biomarkers between a- and a + participants

We compared A− and A + participants using fold-change analyses (Fig. 2). In CU participants, all CSF and plasma pTau biomarkers were elevated in A + individuals relative to A− (Fig. 2A), with mean fold changes ranging from 1.02 to 1.08. In CI participants, CSF pTau biomarkers were similarly higher in A + relative to A− participants (Fig. 2B), with slightly larger fold changes overall (range: 1.05–1.14). Across both CU and CI groups, plasma pTau217, CSF pTau217 and plasma BD-pTau217 consistently exhibited the highest mean fold-changes.

**Figure 2:**
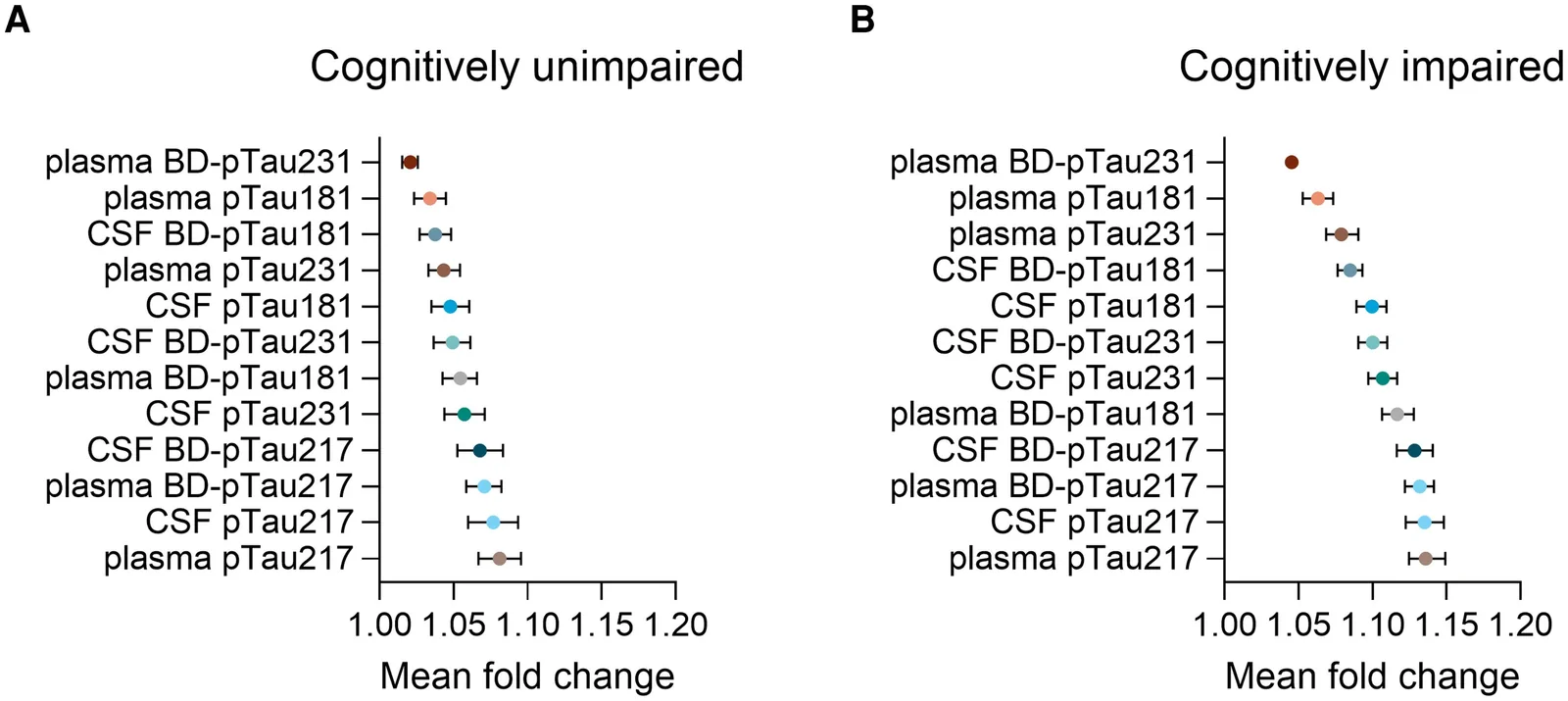
**Fold change of tau biomarkers in CU and CI participants.** (**A**) Fold change and 95% bootstrapped CI of CSF and plasma pTau biomarkers in cognitively unimpaired participants (CU A− versus CU A+). (**B**) Fold change of CSF and plasma pTtau biomarkers in cognitively impaired participants (CI A− versus CI A+). Fold change values >1 indicate higher levels in A + participants relative to A− participants.

### Association between amyloid-β burden and pTau biomarkers

We next assessed the relationship between pTau biomarkers and amyloid-β burden. Spearman correlations revealed significant positive associations for all biomarkers in both CSF and plasma (Fig. 3). Plasma BD-pTau217 showed the strongest correlation with amyloid-PET (ρ = 0.74, *P* = 1.3e-158). CSF and CSF BD measurements were similar, differing by only ∼0.01 in rho across the different analytes. Plasma BD assays showed better correlations with Centiloids than conventional plasma pTau assays across analytes. Notably, among participants with very low amyloid-β (Centiloid ∼0), plasma BD-pTau showed minimal dispersion compared to conventional plasma pTau biomarkers. The relationship between pTau biomarkers and amyloid-β burden in CU and CI subgroups is shown in the Supplementary Materials (Supplementary Fig. 1). We additionally evaluated the correspondence between CSF and plasma measurements for each pTau analyte. CSF-plasma associations were moderate to strong and were generally stronger in BD compared to conventional assays (Supplementary Fig. 2).

**Figure 3:**
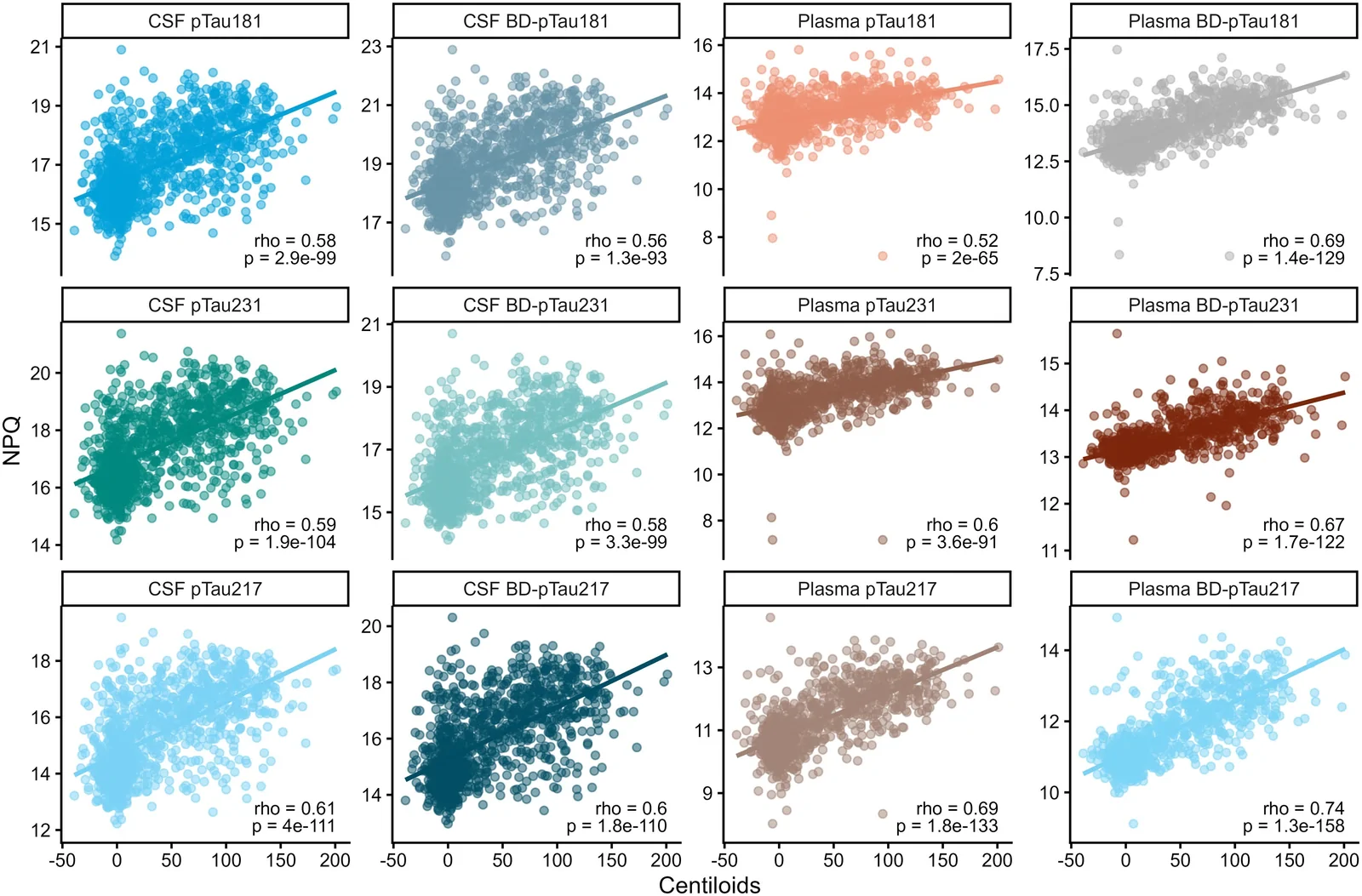
**Relationship between CSF and plasma pTau biomarkers and amyloid-β burden.** Scatterplots showing individual participants’ biomarker values as a function of amyloid-β burden (Centiloids). Linear regression lines are fitted for each biomarker, and panels are faceted by biomarker, ordered by analyte (pTau181, pTau231, pTau217) and sample type (CSF, CSF BD, Plasma, Plasma BD). Spearman’s rank correlation coefficients (ρ) and *P*-value are displayed in each panel.

### Diagnostic performance of conventional versus BD-pTau biomarkers

To evaluate diagnostic performance, we performed ROC analyses, with the AUC calculated for each biomarker for predicting amyloid-PET positivity in all participants, as well as in CU and CI subgroups (Fig. 4).

**Figure 4:**
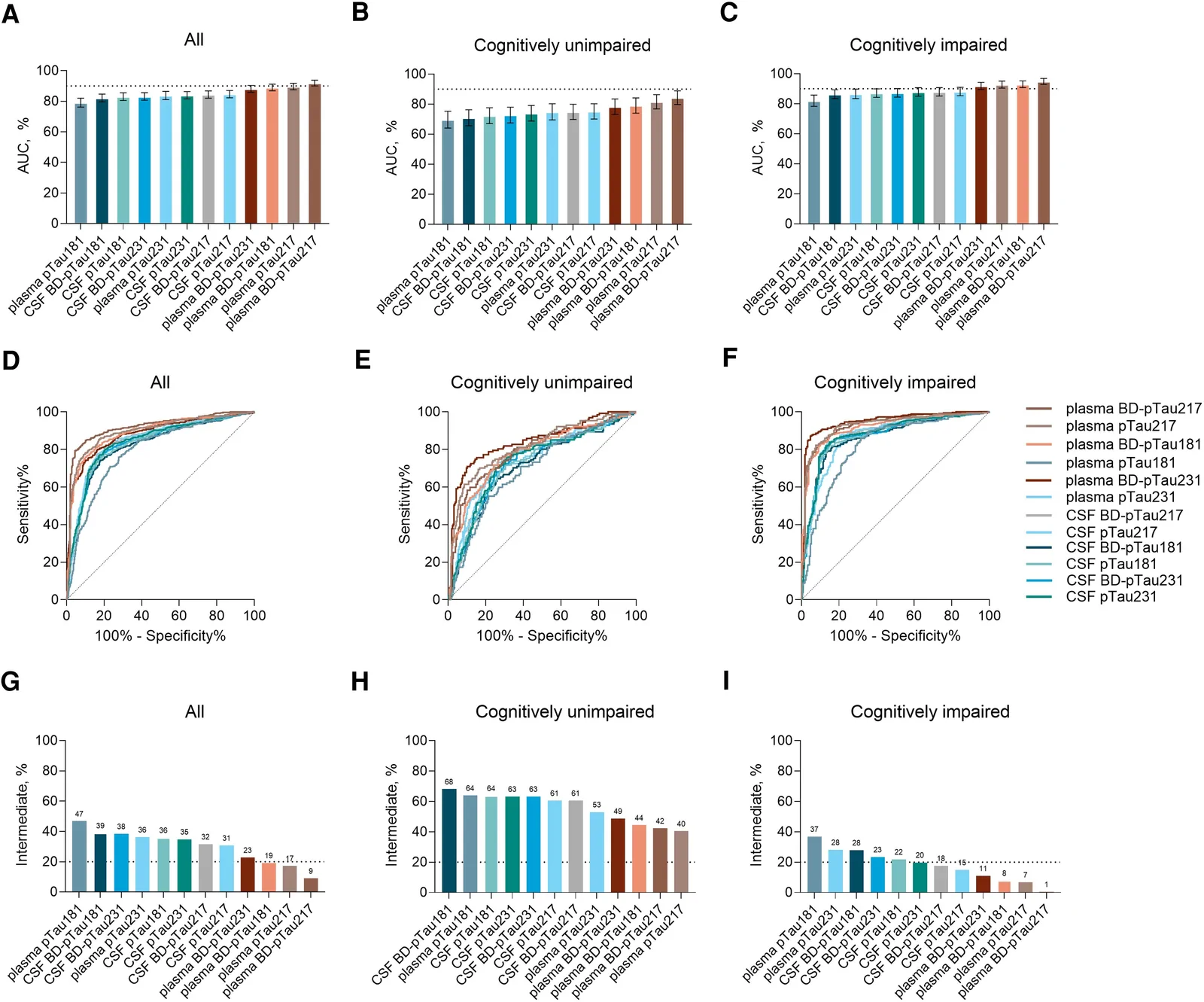
**Performance of CSF and plasma pTau biomarkers in discriminating amyloid-β status across diagnostic groups.** Barplots showing the area under the curve for each biomarker across (**A**) all participants, (**B**) cognitively unimpaired participants, and (**C**) cognitively impaired participants. Error bars represent 95% confidence intervals. The dashed lines at 90% AUC are arbitrary and presented to aid in visual interpretation. ROC curves are shown for (**D**) all participants, (**E**) cognitively unimpaired participants, and (**F**) cognitively impaired participants, illustrating sensitivity versus 1-specificity for each biomarker. AUC values and 95% confidence intervals are provided in Supplementary Table 1. Using a three-range method corresponding to 90% sensitivity and 90% specificity, the proportion of participants classified in the intermediate range is shown for (**G**) all participants, (**H**) cognitively unimpaired participants, and (**I**) cognitively impaired participants. The dashed lines in these panels indicate the clinically relevant threshold of 20%, i.e. the recommended maximum proportion of participants classified in the intermediate range for clinical use.

In the entire sample (Fig. 4A), AUC values ranged from 79.1 to 91.9. Plasma BD-pTau217 had the highest AUC (AUC = 91.9, 95% CI: 90.02–93.8), followed by plasma pTau217 (AUC = 89.7, 95% CI: 87.5–91.8), plasma BD-pTau181 (AUC = 89.0, 95% CI: 86.9–91.2) and plasma BD-pTau231 (AUC = 88.1, 95% CI: 85.9–90.4). Similar results were observed in CU individuals, with overall lower AUCs (AUC values ranging from 69.7 to 84.3—Fig. 4B). Finally, in CI individuals, AUC values ranged from 82.1 to 94.9. We found that plasma BD-pTau217 (AUC = 94.9, 95% CI: 92.9–96.9) and plasma BD-pTau181 (AUC = 93.0, 95% CI: 90.8–95.2) had the highest AUCs, followed by plasma pTau217 (AUC = 92.8, 95% CI:90.4–95.1) (Fig. 4C). Plasma pTau181 had the lowest AUC in all three groups. All AUC values and 95% CI are reported in Supplementary Table 1. The corresponding ROC curves for the entire sample, CU and CI groups are shown in Fig. 4D–F. To compare the performance of BD assays versus conventional assays, we computed pairwise comparisons using DeLong’s test—*P*-values are provided in Supplementary Table 2. Overall, plasma BD assays showed clear advantages over conventional assays for all plasma biomarkers (Fig. 5), with the greatest improvements observed for plasma pTau181 (all sample: AUC difference = 9.92, *P* = 1.32e-17; CU: AUC difference = 9.40, *P* = 1.74e-05; CI: AUC difference = 10.9, *P* = 1.73e-10) and plasma pTau231 (all sample: AUC difference = 4.36, *P* = 1.12e-05; CU: AUC difference = 3.46, *P* = 0.0931; CI: AUC difference = 5.27, *P* = 0.000122). In CSF, BD-pTau181 (all sample: AUC difference = 0.886, *P* = 2.35e-06; CU: AUC difference = 1.42, *P* = 0.00258; CI: AUC difference = 0.729, *P* = 0.000912) and pTau231 (all sample: AUC difference = 0.819, *P* = 6.00e-05; CU: AUC difference = 1.15, *P* = 0.0153; CI: AUC difference = 0.586, *P* = 0.0126) showed small but significant improvement compared to conventional pTau, while no significant difference was observed between BD and conventional CSF pTau217. Sensitivity, specificity and cutpoints are reported in Table 2. Using amyloid-PET as the reference, none of the plasma or CSF biomarkers achieved both 90% sensitivity and 90% specificity when evaluated with dichotomized classification.

**Figure 5:**
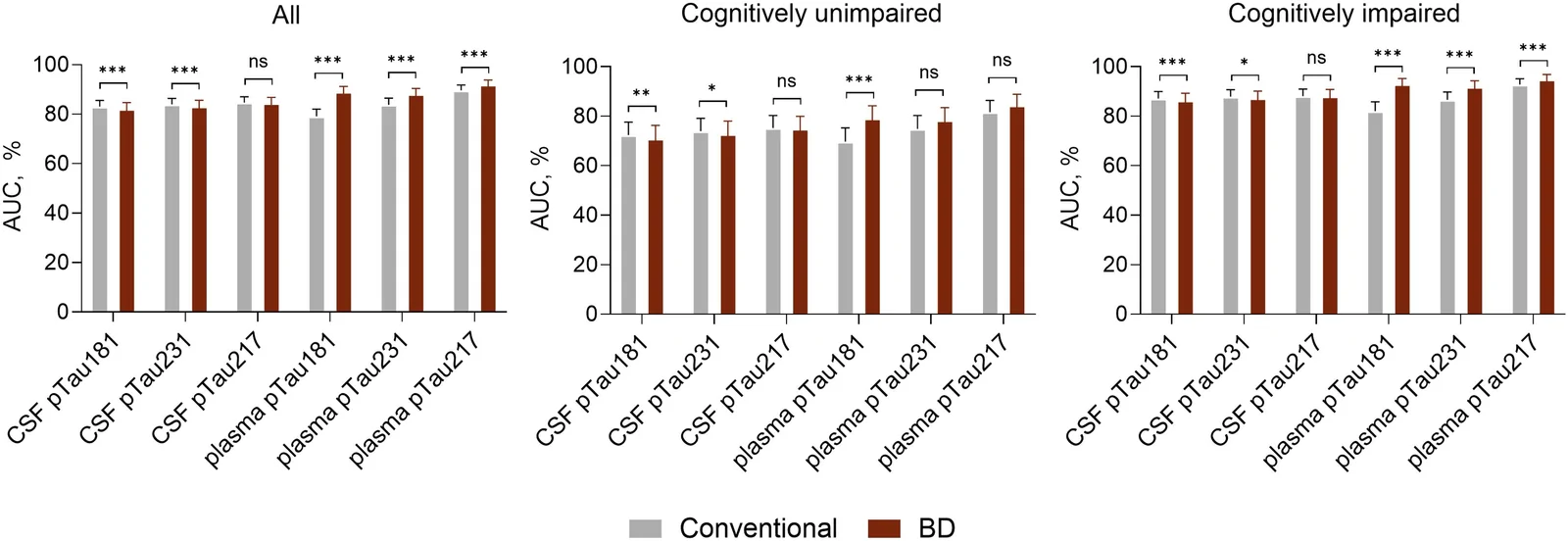
**Comparison of conventional and BD- pTau assays across diagnostic groups.** Area under the ROC curve (AUC) for conventional and BD-pTau assays is shown for all participants, cognitively unimpaired (CU), participants and cognitively impaired (CI) participants. For each group, conventional assays are displayed alongside their BD counterparts to highlight differences in predictive performance. Asterisks indicate statistically significant improvements with BD assays compared to conventional assays, based on Delong’s tests corrected *P*-values using the false discovery rate (* = *P* < 0.05; ** = *P* < 0.01; *** = *P* < 0.001).

**Table 2:** Sensitivity, specificity and optimal cutpoints of pTau biomarkers

	All			Cognitively unimpaired			Cognitively impaired		
	Cutpoint	Sensitivity	Specificity	Cutpoint	Sensitivity	Specificity	Cutpoint	Sensitivity	Specificity
CSF BD-pTau181	18.87	74.2 (70.4–77.7)	82.0 (78.6–85.0)	18.57	64.5 (56.4–72.0)	73.9 (68.6–78.6)	19.07	78.9 (74.6–82.6)	86.5 (81.9–91.2)
CSF BD-pTau217	15.88	77.9 (74.2–81.2)	82.9 (79.5–85.8)	15.53	68.8 (60.7–75.9)	77.0 (71.8–81.4)	15.93	83.8 (79.9–87.1)	84.6 (79.7–88.5)
CSF BD-pTau231	16.54	78.5 (74.8–81.7)	80.2 (76.7–83.3)	16.29	68.1 (60.0–75.2)	74.2 (69.0–78.9)	16.70	83.1 (79.1–86.4)	85.4 (80.6–89.2)
CSF pTau181	17.02	74.0 (70.2–77.5)	83.8 (80.5–86.6)	16.49	71.6 (63.7–78.4)	69.5 (64.0–74.5)	17.02	81.8 (77.8–85.3)	85.4 (80.6–89.2)
CSF pTau217	15.44	77.5 (73.8–80.8)	83.6 (80.3–86.5)	14.84	73.1 (65.2–79.7)	73.9 (68.6–78.6)	15.46	83.8 (79.9–87.1)	85.8 (81–89.5)
CSF pTau231	17.21	80.1 (76.6–83.3)	79.5 (75.9–82.6)	17.04	70.2 (62.2–77.1)	74.6 (69.3–79.2)	17.42	83.8 (79.9–87.1)	85.0 (80.2–88.8)
plasma BD-pTau181	13.91	77.0 (72.9–80.6)	88.8 (85.6–91.4)	13.87	59.8 (51.2–68.0)	88.3 (83.8–91.7)	13.96	82.9 (78.5–86.6)	90.4 (85.7–93.7)
plasma BD-pTau217	11.61	79.4 (75.4–82.8)	95.5 (93.2–97.0)	11.38	70.9 (62.4–78.1)	89.8 (85.5–93.0)	11.60	87.1 (83.1–90.3)	95.2 (91.4–97.4)
plasma BD-pTau231	13.38	74.4 (70.2–78.1)	89.9 (86.8–92.3)	13.23	75.6 (67.4–82.2)	72.7 (66.9–77.8)	13.38	82.9 (78.5–86.6)	90.0 (85.1–93.3)
plasma pTau181	13.13	75.9 (71.8–79.6)	72.3 (68.0–76.1)	13.21	55.1 (46.4–63.5)	78.5 (73.1–83.1)	13.04	85.9 (81.7–89.2)	68.4 (61.8–74.3)
plasma pTau217	11.31	78.0 (74.0–81.6)	90.1 (87.1–92.5)	11.10	70.1 (61.6–77.4)	83.2 (78.1–87.3)	11.49	82.3 (77.8–86.0)	92.3 (87.9–95.2)
plasma pTau231	13.31	77.8 (73.8–81.4)	80.65 (76.8–84.0)	13.32	60.6 (51.9–68.7)	82.8 (77.7–86.9)	13.27	86.8 (82.7–90.0)	76.1 (69.9–81.4)

This table shows the sensitivity, specificity and optimal cutpoint that best discriminate A- and A + participants for each pTau biomarker determined using the Youden index.

Using a three-range classification method based on cutpoints corresponding to 90% sensitivity and specificity, we computed the proportion of participants falling in the intermediate range for each biomarker (Fig. 4G–I; Table 3). Among CU participants, the proportion of individuals in the intermediate range was substantially higher, ranging from 40 to 68%. In CI participants, intermediate proportions ranged from 1 to 37%, and across all participants, the range was 9 to 47%. Across all participants and in CI individuals, plasma BD-pTau181, plasma pTau217 and plasma BD-pTau217 consistently had <20% of participants in the intermediate range, meeting the recommended threshold for clinical applicability. Sensitivity analyses using 95% sensitivity/specificity thresholds were more stringent: only plasma BD-pTau217 in CI remained below 20% intermediate, whereas no biomarkers met this criterion in CU or across all participants (Supplementary Table 3).

**Table 3:** Cutpoints and proportion of participants classified in the intermediate range for each biomarker using 90% sensitivity and specificity thresholds

Biomarker	All		Cognitively unimpaired		Cognitively impaired	
	% intermediate	Cutpoints	% intermediate	Cutpoints	% intermediate	Cutpoints
CSF BD-pTau181	39.0	18.1–19.4	68.1	17.6–19.5	28.0	18.3–19.4
CSF BD-pTau217	31.6	15.0–16.5	60.6	14.5–16.7	17.7	15.3–16.4
CSF BD-pTau231	38.4	15.8–17.3	63.3	15.5–17.3	23.4	15.9–17.0
CSF pTau181	36.0	16.2–17.5	63.8	15.6–17.5	21.9	16.4–17.4
CSF pTau217	30.8	14.4–16.0	60.6	13.8–16.1	15.0	14.8–15.8
CSF pTau231	34.9	16.5–18.1	63.3	16.0–18.2	19.8	16.7–17.8
plasma BD-pTau181	19.2	13.4–13.9	44.4	13.1–13.9	7.93	13.7–13.9
plasma BD-pTau217	9.08	11.2–11.4	42.3	10.8–11.4	0.554	11.4–11.5
plasma BD-pTau231	22.8	13.2–13.4	48.8	13.1–13.4	11.1	13.3–13.4
plasma pTau181	46.8	12.7–13.6	64.0	12.3–13.5	36.9	12.9–13.7
plasma pTau217	17.4	10.8–11.3	40.5	10.5–11.3	6.83	11.1–11.4
plasma pTau231	36.3	12.9–13.7	53.0	12.6–13.6	28.2	13.1–13.8

In exploratory head-to-head analyses (*N* = 142), all plasma pTau217 assays demonstrated high diagnostic accuracy (Supplementary Fig. 3; Supplementary Table 4; Supplementary Table 5). Similar results were obtained in CU and CI subsamples.

## Discussion

In this study, we systematically compared conventional and BD-pTau biomarkers across CSF and plasma for their relationship with amyloid-β pathology and their diagnostic performance for AD. Across all analytes, A + participants consistently exhibited higher biomarker levels than A− participants, including among CU individuals, highlighting the sensitivity of pTau to early AD-related changes. CSF BD-pTau assays closely mirrored conventional CSF pTau measurements, suggesting that enrichment for CNS-derived tau does not substantially alter the signal in a fluid already in direct contact with the brain. In plasma, BD-pTau assays showed improved correlations with amyloid-β burden compared with conventional plasma pTau, particularly in individuals with very low amyloid-β, and generally demonstrated higher AUCs for distinguishing elevated amyloid-PET across the overall cohort as well as in CU and CI subgroups. Notably, plasma BD-pTau217 and BD-pTau181 showed the strongest diagnostic performance, whereas conventional plasma pTau181 consistently showed the lowest. Despite these improvements, none of the biomarkers reached the 90% sensitivity and specificity benchmark. However, a three-range method based on 90% sensitivity and specificity cutpoints enabled plasma BD-pTau217, plasma pTau217 and plasma BD-pTau181 to meet recommended potential clinical applicability thresholds by limiting the intermediate range to <20% of participants.

First, we observed that both conventional and BD-pTau measured in CSF showed highly similar associations with amyloid-β burden measured with PET. This close correspondence is biologically expected, as CSF is in direct contact with the central nervous system and is therefore inherently enriched for CNS-derived tau species. As a result, both CSF BD and conventional BD assays are likely capturing overlapping components of AD-related tau biology, particularly in relation to upstream amyloid-β pathology. The similarity in their relationships with Centiloids supports the notion that BD-pTau reflects a common CNS-driven signal across measurement contexts, reinforcing the biological validity of the assay and its relevance for characterizing AD pathology. Interestingly, in plasma, the correlations with amyloid-β burden improved when using BD-pTau measurements, suggesting that brain-derived assays may offer the greatest advantage in peripheral biofluids, where the signal from CNS tau is otherwise diluted by peripheral sources. These findings suggest that the added value of BD enrichment may be most evident in peripheral biofluids, whereas CSF-based measures already provide a relatively direct window into CNS AD-related pathology.

Building on this, the moderate plasma-CSF correlations observed for the same analyte further underscore that these two compartments, while reflecting the same underlying brain pathology, are shaped by fundamentally different biological and analytical factors. These distinct clearance kinetics and sources of variability mean that even a perfectly brain-specific assay will not produce a 1:1 correspondence across compartments. Interestingly, these same factors may explain why plasma BD-pTau217 numerically matched or exceeded CSF measures for binary classification of amyloid-β even without BD enrichment (although no formal statistical comparison between plasma and CSF performance was performed in this study). First, CSF collection introduces well-documented preanalytical variability from tube adsorption, volume effects, freeze-thaw sensitivity and procedural differences that plasma largely avoids with modern high-sensitivity platforms.^22,23^ Second, because pTau217 enters the bloodstream by crossing the blood-brain barrier at capillaries that perfuse every brain region, plasma effectively aggregates contributions from the entire brain into one measurement, providing a globally integrated measure of tau phosphorylation rather than a signal shaped by local drainage dynamics.^24^ In contrast, CSF concentrations are shaped by compartment-specific dynamics, including glymphatic clearance efficiency, CSF turnover rate (which declines ∼50% with ageing) and rostro-caudal concentration gradients between ventricular and lumbar compartments—all of which introduce measurement variability unrelated to the actual burden of amyloid-driven tau pathology.^25,26^ Third, CSF pTau217 concentrations, despite their larger absolute fold-change, appear to plateau at higher levels of amyloid-β burden, potentially compressing the dynamic range and widening the indeterminate zone among individuals with moderate-to-advanced pathology.^27^ Plasma pTau217, while showing smaller absolute concentration differences, continues to rise more linearly across the disease trajectory, preserving classificatory separation even at advanced stages where CSF values may cluster.^28^ Thus, while CSF provides a more direct window into CNS pathology—as evidenced by the similar results of BD and conventional measures in that matrix—the combination of brain-derived specificity, whole-brain signal integration, large dynamic range and reduced preanalytical noise may give plasma BD-pTau217 a classificatory advantage, precisely because the BD enrichment strategy most effectively removes the peripheral noise that is uniquely problematic in blood. Further investigation across independent cohorts and platforms will be needed to fully elucidate the biological and analytical factors driving these compartment-specific differences in classificatory performance.

Overall, the strongest sensitivity and specificity for BD-pTau were observed in participants with cognitive impairment, which is biologically plausible given that these individuals are likely to harbour higher levels of AD pathology,^29^ making detection more reliable. This pattern aligns with findings from recent reviews and meta-analyses of blood-based phosphorylated tau biomarkers, which consistently report improved diagnostic performance in symptomatic populations.^4^ While these findings are encouraging, diagnostic performance was more modest in cognitively unimpaired individuals, underscoring the need for additional studies to better define the utility of BD-pTau in preclinical or asymptomatic populations.

International guidelines, including recommendations from the World Health Organization,^30^ the Global CEO initiative^31^ and the Alzheimer’s Association,^32^ suggest that blood-based biomarkers should achieve 90% sensitivity and specificity in their intended-use populations to be considered suitable for clinical diagnostic application. This benchmark is based on the performance of FDA-approved CSF biomarker assays, which reliably detect amyloid-β pathology with roughly 90% accuracy compared with visual reads of amyloid-PET.^33^ In our study, although BD-pTau showed good discrimination, the 90% sensitivity and specificity threshold was not reached in CI participants when using dichotomized biomarker values. Nevertheless, when applying a three-range classification approach based on 90% sensitivity and specificity cutpoints, plasma BD-pTau217, plasma pTau217 and plasma BD-pTau181 met recommended criteria for potential clinical applicability by yielding an intermediate range comprising <20%^31,34^ of participants in CI individuals. Notably, while plasma pTau217 already performed strongly using this framework (7% intermediate), enrichment for BD-pTau further reduced the intermediate range to 1% for plasma BD-pTau217. Both CSF pTau217 and CSF BD-pTau217 also remained below the 20% threshold, indicating robust performance irrespective of assay format. In contrast, the greatest relative gains with BD enrichment were observed for plasma pTau181 and pTau231, where the proportion of individuals in the intermediate range was markedly reduced (from 37% to 8% and from 28% to 11%, respectively), whereas corresponding improvements in CSF were more modest. Collectively, these findings indicate that BD enrichment substantially enhances the clinical interpretability of plasma pTau biomarkers—particularly pTau181 and pTau231, which have shown more limited diagnostic performance in prior studies—by reducing intermediate uncertainty and enabling these assays to meet consensus-recommended criteria in three-range classification frameworks. However, while these results are promising, they should be interpreted cautiously in the absence of external validation and independent cohort replication.

In exploratory head-to-head analyses (*N* = 142), plasma BD-pTau217 showed broadly comparable diagnostic performance to commercially available immunoassays and IPMS platforms. While BD improved the performance of plasma pTau217 in our main analyses, its advantage was less apparent in this small subsample, and AUCs were similar across assays. Given the limited sample size and associated reduction in statistical power, these findings should be interpreted as exploratory and considered hypothesis-generating rather than definitive. Larger, adequately powered head-to-head studies will be required to determine whether BD enrichment confers consistent performance advantages across assay platforms.

Although BD-pTau assays demonstrated superior discrimination between A− and A + individuals compared with conventional assays, the magnitude of the group separation, as reflected by fold-change analyses, was largely similar for both CSF and plasma pTau217 across BD and conventional measures. This apparent discrepancy likely reflects differences in within-group variability rather than differences in between-group effect size. BD assays showed lower conventional deviation within diagnostic groups, particularly in plasma, resulting in reduced overlap between A− and A + individuals. Consequently, even when mean differences were comparable, BD assays achieved improved classification performance by limiting extreme values and reducing the likelihood of misclassification at the individual level. These findings suggest that enrichment for CNS-derived tau enhances measurement precision and specificity rather than amplifying biological contrast between amyloid-defined groups. From a clinical perspective, reduced variability and overlap are critical for minimizing misclassification and improving the reliability of blood-based pTau assays in real-world diagnostic and triage settings.

While conventional plasma and CSF pTau assays have been extensively studied in their association with amyloid-β and tau pathology,^35^ it remains unclear how BD-pTau measures relate to tau accumulation. Tracking tau accumulation is critical in AD, as tau burden closely drives rates of clinical and biological progression in A + individuals.^36,37^ While our results begin to address this gap by demonstrating that BD assays improve discrimination between A− and A + participants, future work should assess whether BD-pTau measurements can serve as a more precise proxy for CNS tau pathology and facilitate earlier identification of biologically at-risk individuals in both research and clinical settings. In this connection, it will also be important for future studies to assess the relationship between BD-pTau and fluid measures closely associated with tau pathology such as MTBR-243.^38,39^ It is hypothesized that a combination of fluid measurements will permit for the biological staging of AD,^29,40,41^ which would circumvent the need for multiple PET scans as required by the current AD staging framework.^42,43^

Several limitations should be considered when interpreting our findings. First, our study included highly motivated participants who were willing to undergo both CSF collection and detailed clinical assessments, which may limit generalizability to broader clinical populations. Second, biomarker measurements may be influenced by pre-analytical factors such as freeze–thaw cycles, which could affect protein stability and assay signal, though we minimized this by careful aliquoting. Third, the BD-pTau results were generated using the NULISA research platform, and their performance may not directly generalize to emerging fully automated IVD platforms used in routine clinical settings, which may differ in assay design, analytical sensitivity, and pre-analytical handling. Fourth, while brain-derived pTau enrichment improves CNS specificity, the approach remains relatively new and requires further validation across independent cohorts and clinical settings. Importantly, because BD-pTau assays are not yet widely available across established research cohorts, we were unable to include independent replication sample, and external validation of our findings will be essential. Fifth, we could not assess the potential impact of peripheral factors such as neuropathy or kidney dysfunction on BD-pTau measurements. Therefore, in specific population (e.g. ALS, peripheral neuropathy, etc.), it is plausible that BD-pTau may performed even better compared to conventional pTau. Sixth, although plasma BD-pTau showed promising correlations with amyloid-PET, we cannot yet determine its utility for predicting longitudinal disease progression. Finally, while our study evaluated three pTau analytes, other emerging phospho-tau epitopes or multi-marker panels may provide complementary information that was not captured here. Together, these limitations highlight the need for larger, longitudinal, and multi-centre studies to fully establish the clinical performance and generalizability of BD-pTau assays.

## Supplementary Material

fcag376_Supplementary_Data

## Data Availability

Data used in this study were obtained from the Alzheimer’s Disease Neuroimaging Initiative (ADNI) database (http://adni.loni.usc.edu). ADNI data are publicly available to qualified researchers following registration and approval through the ADNI data access process. A full list of participating ADNI investigators and institutions can be found at https://adni.loni.usc.edu/about/. The analyses conducted in this study did not require the development of custom code, as all procedures were performed using standard statistical methods.
