# Supplementary material for: Comparison of brain-derived versus conventional pTau biomarkers in CSF and plasma for detecting Alzheimer’s disease
Source: Brain Commun. 2026 Oct 7;8(5):fcag376. doi: 10.1093/braincomms/fcag376 (PMC13669629; doi:10.1093/braincomms/fcag376)
Supplement: Supplementary file 1 [file fcag376_supplementary_data.pdf]

## Supplementary Materials

**Trudel, Therriault, Macedo et al.**

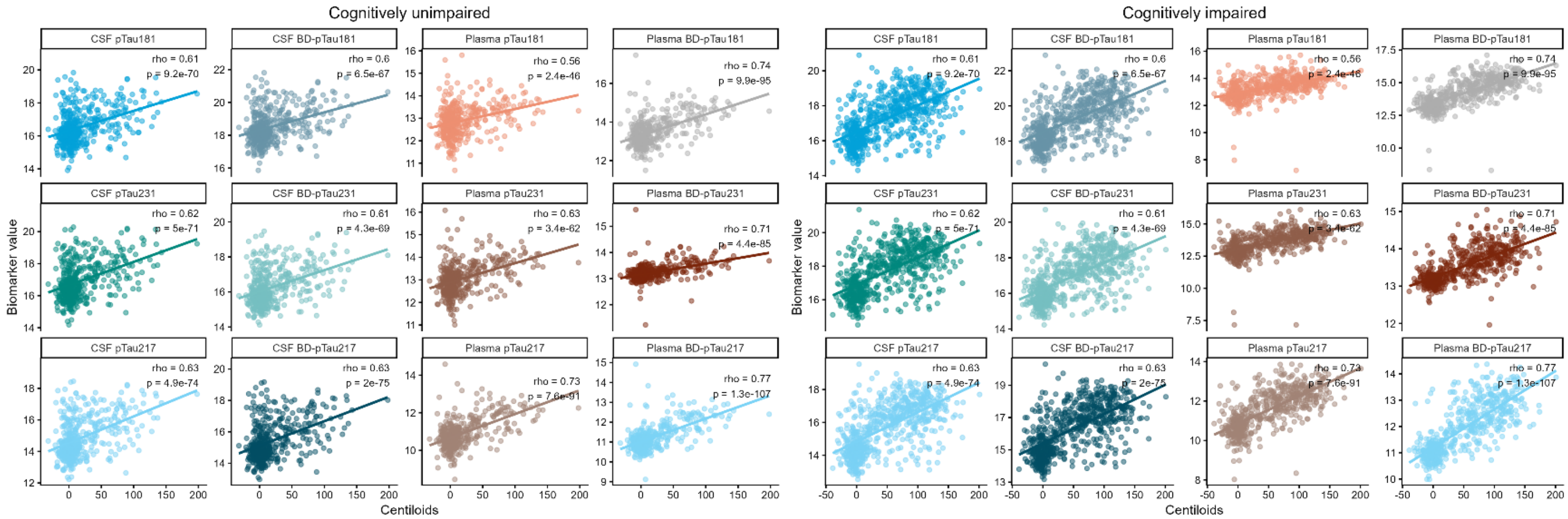


**Supplementary Figure 1. Relationship between CSF and plasma pTau biomarkers and amyloid-β burden in CU and CI subgroups.** Scatterplots show individual participants' biomarker values as a function of amyloid-β burden (Centiloids). Linear regression lines are fitted for each biomarker, and panels are faceted by biomarker, ordered by analyte (pTau181, pTau231, pTau217) and sample type (CSF, CSF BD, Plasma, Plasma BD).

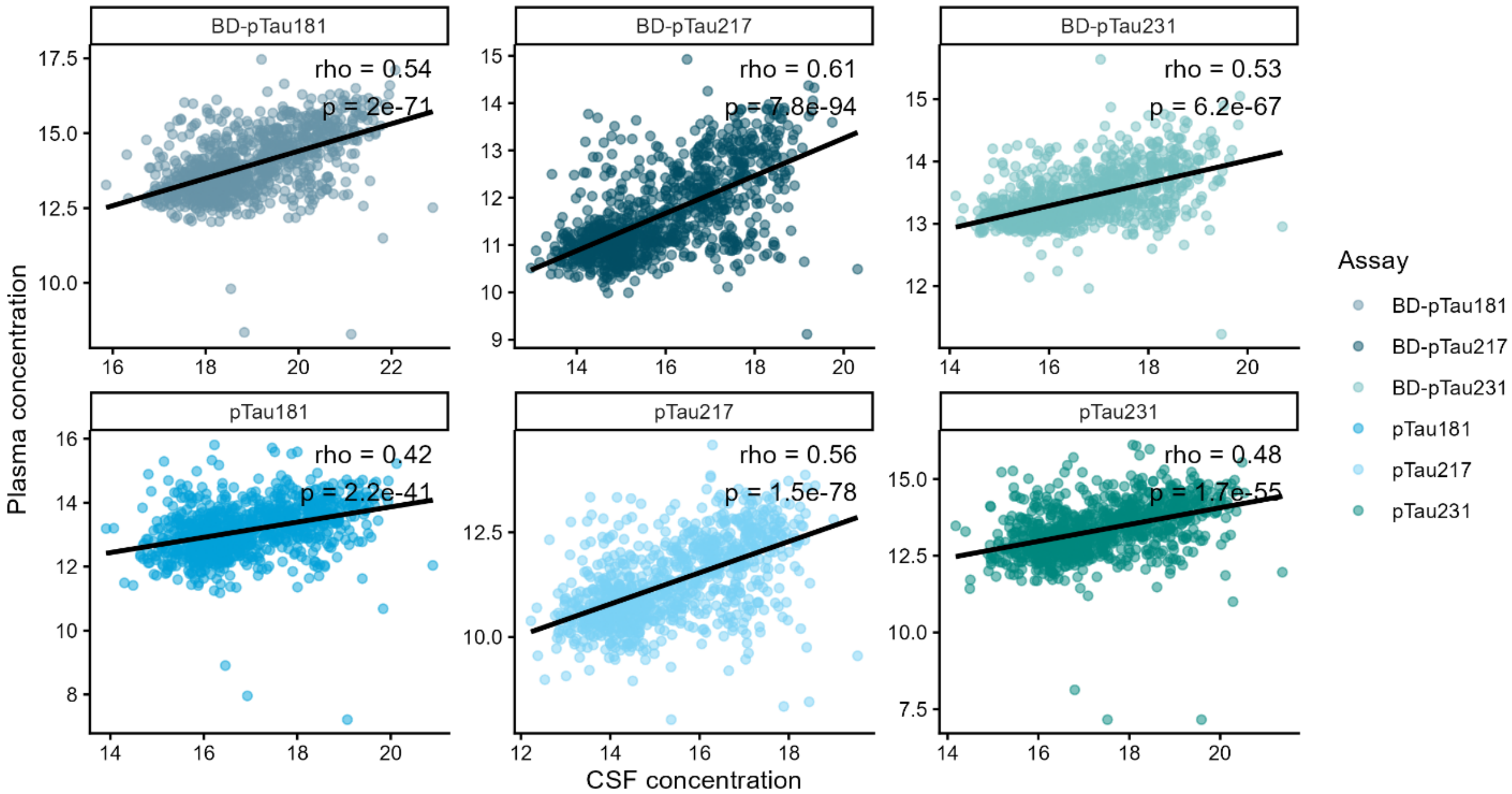


**Supplementary Figure 2. Relationship between CSF and plasma pTau biomarkers.** Scatterplots show individual participants' plasma concentration as a function of CSF concentration. Linear regression lines are fitted for each biomarker, and panels are faceted by analyte (pTau181, pTau231, pTau217) and sample type (conventional or brain-derived).

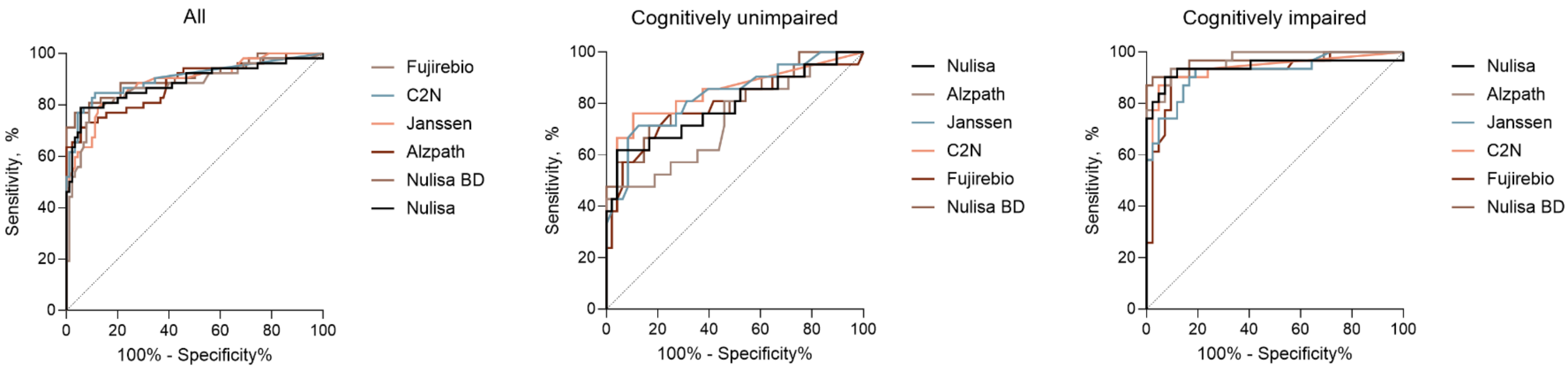


**Supplementary Figure 3. ROC curves illustrating sensitivity versus 1-specificity for different plasma pTau217 assays for all participants, cognitively unimpaired participants and cognitively impaired participants.** AUC values are listed in Supplementary Table 4.

| Biomarker | All<br>AUC, 95% CI | Cognitively unimpaired<br>AUC, 95% CI | Cognitively impaired<br>AUC, 95% CI |
|---|---|---|---|
| Plasma pTau181 | 79.1 (76.2–82.1) | 69.7 (64.1–75.3) | 82.1 (78.3–85.8) |
| CSF BD-pTau181 | 82.1 (79.6–84.7) | 71.0 (65.7–76.3) | 86.4 (83.5–89.3) |
| CSF pTau181 | 83.0 (80.5–85.5) | 72.4 (67.2–77.7) | 87.1 (84.3–90.0) |
| CSF BD-pTau231 | 83.2 (80.7–85.6) | 72.8 (67.6–78.0) | 87.3 (84.4–90.1) |
| Plasma pTau231 | 83.8 (81.2–86.4) | 74.9 (69.5–80.3) | 86.6 (83.4–89.8) |
| CSF pTau231 | 84.0 (81.6–86.4) | 74.0 (68.8–79.1) | 87.9 (85.1–90.7) |
| CSF BD-pTau217 | 84.4 (82.0–86.8) | 74.9 (69.9–80.0) | 88.0 (85.2–90.8) |
| CSF pTau217 | 84.7 (82.3–87.1) | 75.2 (70.1–80.3) | 88.2 (85.4–91.0) |
| Plasma BD-pTau231 | 88.1 (85.9–90.4) | 78.4 (73.2–83.5) | 91.9 (89.5–94.3) |
| Plasma BD-pTau181 | 89.0 (86.9–91.2) | 79.1 (74.0–84.2) | 93.0 (90.8–95.2) |
| Plasma pTau217 | 89.7 (87.5–91.8) | 81.6 (76.9–86.4) | 92.8 (90.4–95.1) |
| Plasma BD-pTau217 | 91.9 (90.0–93.8) | 84.3 (79.8–88.9) | 94.9 (92.9–96.9) |

**Supplementary Table 1. Area under the receiver operating characteristic curves (AUC) for CSF and plasma pTau biomarkers.** AUCs and 95% confidence intervals (CIs) for discrimination of amyloid-positive from amyloid-negative participants are shown for the overall sample, cognitively unimpaired (CU) participants, and cognitively impaired (CI) participants.

| All | | Cognitively unimpaired | | Cognitively impaired | |
|---|---|---|---|---|---|
| ***Comparison*** | ***p-value*** | ***Comparison*** | ***p-value*** | ***Comparison*** | ***p-value*** |
| CSF BD-pTau181 vs pTau181 | **2.35e-06** | CSF BD-pTau181 vs pTau181 | **0.00258** | CSF BD-pTau181 vs pTau181 | **0.000912** |
| CSF BD-pTau231 vs pTau231 | **6.00e-05** | CSF BD-pTau231 vs pTau231 | **0.0153** | CSF BD-pTau231 vs pTau231 | **0.0126** |
| CSF BD-pTau217 vs pTau217 | 0.0910 | CSF BD-pTau217 vs pTau217 | 0.382 | CSF BD-pTau217 vs pTau217 | 0.455 |
| Plasma BD-pTau181 vs pTau181 | **1.32e-17** | Plasma BD-pTau181 vs pTau181 | **1.74e-05** | Plasma BD-pTau181 vs pTau181 | **1.73e-10** |
| Plasma BD-pTau231 vs pTau231 | **1.12e-05** | Plasma BD-pTau231 vs pTau231 | 0.0931 | Plasma BD-pTau231 vs pTau231 | **0.000122** |
| Plasma BD-pTau217 vs pTau217 | **8.00e-05** | Plasma BD-pTau217 vs pTau217 | 0.0571 | Plasma BD-pTau217 vs pTau217 | **0.000871** |

**Supplementary Table 2. Comparison of brain-derived and conventional pTau assays.** Pairwise comparison of diagnostic performance (AUC) between brain-derived and conventional pTau assays in CSF and plasma using DeLong's test. Bold values indicate statistically significant differences after FDR correction ($q < 0.05$). Analyses were conducted in the overall sample, as well as in cognitively unimpaired and cognitively impaired subgroups.

| | All | | Cognitively unimpaired | | Cognitively impaired | |
|---|---|---|---|---|---|---|
| **Biomarker** | % intermediate | Cutpoints | % intermediate | Cutpoints | % intermediate | Cutpoints |
| **CSF BD-pTau181** | 68.3 | 17.7–20.2 | 83.3 | 17.3–20.2 | 54.7 | 18.0–20.1 |
| **CSF BD-pTau217** | 63.3 | 14.5–17.5 | 79.1 | 14.2–17.5 | 53.3 | 14.8–17.4 |
| **CSF BD-pTau231** | 64.0 | 15.5–17.9 | 81.0 | 15.1–17.9 | 52.1 | 15.7–17.9 |
| **CSF pTau181** | 66.1 | 15.7–18.3 | 81.7 | 15.4–18.3 | 53.9 | 16.0–18.3 |
| **CSF pTau217** | 63.2 | 13.9–16.8 | 78.9 | 13.5–16.8 | 50.9 | 14.2–16.8 |
| **CSF pTau231** | 63.3 | 16.0–18.7 | 79.6 | 15.6–18.7 | 50.8 | 16.3–18.7 |
| **plasma BD-pTau181** | 41.5 | 13.1–14.3 | 72.8 | 12.7–14.3 | 24.7 | 13.4–14.3 |
| **plasma BD-pTau217** | 31.2 | 11.0–11.6 | 57.7 | 10.7–11.6 | 13.8 | 11.2–11.6 |
| **plasma BD-pTau231** | 45.1 | 13.1–13.5 | 73.6 | 13.0–13.5 | 29.0 | 13.2–13.5 |
| **plasma pTau181** | 68.5 | 12.4–13.9 | 80.4 | 12.1–13.8 | 65.5 | 12.6–14.0 |
| **plasma pTau217** | 40.1 | 10.5–11.7 | 62.4 | 10.3–11.6 | 24.0 | 10.8–11.7 |
| **plasma pTau231** | 60.2 | 12.6–14.0 | 76.5 | 12.3–13.9 | 52.6 | 12.8–14.1 |

**Supplementary Table 3. Cutpoints and proportion of participants classified in the intermediate range for each biomarker using 95% sensitivity and specificity thresholds.**

| | All | Cognitively unimpaired | Cognitively impaired |
|---|---|---|---|
| ***Assay*** | **AUC, 95% CI** | **AUC, 95% CI** | **AUC, 95% CI** |
| BD-Nulisa | 90.9 (85.2 – 96.6) | 81.4 (69.3 – 93.5) | 96.6 (92.0 – 1) |
| Alzpath | 88.1 (81.9 – 94.4) | 73.5 (59.6 – 87.4) | 96.8 (93.4 – 1) |
| C2N | 90.4 (84.6 – 96.2) | 84.7 (73.2 – 96.1) | 94.0 (88.1 – 99.9) |
| Fujirebio | 87.4 (80.7 – 94.1) | 79.4 (66.1 – 92.6) | 92.4 (85.6 – 99.2) |
| Janssen | 88.9 (83.0 – 94.8) | 83.3 (72.1 – 94.6) | 92.3 (85.7 – 98.8) |
| Nulisa | 88.5 (81.9 – 95.2) | 79.2 (65.9 – 92.4) | 94.3 (84.5 – 1) |

**Supplementary Table 4. Area under the receiver operating characteristic (ROC) curves for plasma pTau217 assays.** AUCs were calculated for the discrimination of amyloid-positive from amyloid-negative participants in the overall sample and separately in cognitively unimpaired (CU) and cognitively impaired (CI) participants, as illustrated in Supplementary Figure 3.

| All | | Cognitively unimpaired | | Cognitively impaired | |
|---|---|---|---|---|---|
| ***Comparison*** | ***p-value*** | ***Comparison*** | ***p-value*** | ***Comparison*** | ***p-value*** |
| BD-Nulisa vs Alzpath | 0.416 | BD-Nulisa vs Alzpath | 0.407 | BD-Nulisa vs Alzpath | 0.952 |
| BD-Nulisa vs C2N | 0.836 | BD-Nulisa vs C2N | 0.622 | BD-Nulisa vs C2N | 0.528 |
| BD-Nulisa vs Fujirebio | 0.416 | BD-Nulisa vs Fujirebio | 0.622 | BD-Nulisa vs Fujirebio | 0.437 |
| BD-Nulisa vs Janssen | 0.532 | BD-Nulisa vs Janssen | 0.622 | BD-Nulisa vs Janssen | 0.437 |
| BD-Nulisa vs Nulisa | 0.416 | BD-Nulisa vs Nulisa | 0.622 | BD-Nulisa vs Nulisa | 0.437 |

**Supplementary Table 5. Exploratory analyses comparing of plasma BD-pTau217 and other plasma pTau217 assays in subsample (N = 142)**. Pairwise comparison of diagnostic performance (AUC) between BD-pTau217 (Nulisa) other plasma pTau217 assays using DeLong's test. Bold values indicate statistically significant differences after FDR correction ($q < 0.05$). Analyses were conducted in the overall sample, as well as in cognitively unimpaired and cognitively impaired subgroups.
